# Bioinformatics Analysis and Functional Verification of ADAMTS9-AS1/AS2 in Lung Adenocarcinoma

**DOI:** 10.3389/fonc.2021.681777

**Published:** 2021-07-29

**Authors:** Wei Liu, Wenguang Luo, Peijie Zhou, Yong Cheng, Liting Qian

**Affiliations:** ^1^Anhui Provincial Hospital, Cheeloo College of Medicine, Shandong University, Jinan, China; ^2^Department of Radiation Oncology, Anhui Provincial Hospital, The First Affiliated Hospital of University of Science and Technology of China, Hefei, China

**Keywords:** ADAMTS9, ADAMTS9-AS1, ADAMTS9-AS2, lung adenocarcinoma, ceRNAs

## Abstract

Long non-coding RNAs (lncRNAs), as competitive endogenous RNAs (ceRNAs), play a critical role in biological processes of cancer. However, the roles of specific lncRNAs in ceRNA network of lung adenocarcinoma (LUAD) remains largely unclear. Herein, we identified the roles of lncRNA ADAMTS9-AS1/AS2 (ADAMTS-AS1/AS2) in lung adenocarcinoma by bioinformatics analyses and functional verification. First, differentially expressed genes ADAMTS9-AS1, ADAMTS9-AS2 and ADAMTS9 were screened out from GSE130779. Then the expression correlation of these three genes was analyzed. The results showed that ADAMTS9-AS1, ADAMTS9-AS2 and ADAMTS9 were down-regulated in LUAD, and were positively correlated with each other. After that, miRcode was used to find miR-150 which binds to ADAMTS9-AS1/ADAMTS9-AS2/ADAMTS9. Next, co-expression analysis and functional enrichment analyses were performed to further analyze differentially expressed genes. The results showed that the differentially expressed genes were mainly enriched in Beta3 integrin cell surface interactions and epithelial-to-mesenchymal transition. Finally, the cell functions of ADAMTS9-AS1 and ADAMTS9-AS2 in A549 and NCI-H1299 cell lines were verified. *In vitro* cell studies confirmed that ADAMTS9-AS1 and ADAMTS9-AS2 play an inhibitory role in LUAD cells.

## Introduction

Lung cancer is the leading cause of cancer-related death (1). Every year, there are 1.8 million people diagnosed with lung disease, and 1.6 million people die from it (2). Lung adenocaricinoma (LUAD), also known as pulmonary adenocarcinoma, is a type of non-small-cell lung carcinoma (NSCLC) and accounts for approximately 40% of all lung cancers (3, 4). Despite great improvements in LUAD research and treatment, the prognosis for LUAD patients remains poor and the mortality rates have not been improved significantly (5). Therefore, in order to improve the prognosis of patients with LUAD, it is urgent to identify the molecular mechanism, which is of great significance to find effective biomarkers for the early diagnosis. Long non-coding RNAs (lncRNAs), as a novel class of noncoding RNAs with more than 200 nucleotides in length, have attracted increasing attention (6, 7). Although lncRNAs do not have the ability to encode proteins, they may play an important role in diverse biological processes related to human cancer (8). For example, lncRNAs are involved in the pathogenesis of many different cancers, including liver cancer (9), colorectal cancer (10), bladder cancer (11), prostate cancer (12), as well as NSCLC (13). Currently, several lncRNAs have been reported to be closely related to clinical relevance, biological-function and potential mechanisms regarding LUAD (14, 15). It is obvious that lncRNAs play an important role in LUAD, whereas the specific role and molecular mechanisms of lncRNAs underlying the initiation and progression of LUAD remain to be explored.

To unveil how lncRNAs exert their diverse biological functions in cancer, a competitive endogenous RNA (ceRNA) hypothesis has been proposed (16). ceRNA hypothesis suggests that mRNAs and lncRNAs can act as natural miRNA “sponges”, and suppress the function of miRNA by competing binding to one or more microRNA (miRNA) response elements in regulatory networks, resulting in pathogenic conditions. ceRNAs compete with other RNAs to bind miRNAs through miRNA response elements, affecting the regulation of miRNAs on target genes and ultimately influencing the proliferation, apoptosis, migration and invasion of various tumors (17). Several studies have indicated that the ceRNA regulation theory was involved in human cancer initiation and progression (18–20), which may provide new insights for the diagnosis and treatment of tumors. Based on ceRNA theory, a study pointed that TTN-AS1 can be used as a ceRNA to bind to MIR-142-5p and indirectly up-regulate the expression of cyclin-dependent kinase 5 (CDK5) in LUAD (21). The research results of Zhu et al. (22) proved that LINC00968 can be used as a combination of ceRNA and miR-21-5p to promote the accumulation of Smad7, the target of miR-21-5p, and then to inhibit the proliferation, migration and invasion of LUAD. LINC00968 can be used as a prognostic biomarker and therapeutic target of LUAD. Coincidentally, a research showed that Linc00662, an oncogene in lung cancer, can be recognized as a ceRNA sponge, regulating miR-145-5p to affect the development of LUAD, which may become a potential target for LUAD therapy (23). Based on the above research results, we hypothesized that lncRNAs may function as ceRNAs in LUAD. Understanding how lncRNAs function as ceRNAs will contribute to revealing the carcinogenesis of LUAD and finding the diagnostic biomarker or therapeutic target of LUAD.

In the present study, through a series of bioinformatics analyses, we found ADAMTS9-AS1/ADAMTS9-AS1 could competitively bind miR-150 to rescue the inhibition of ADAMTS9 by miR-150, and then regulate the migration and invasion of LUAD cells by influencing on Beta3 integrin cell surface interactions or epithelial-to-mesenchymal transition signaling pathway. After that, a series of functional experiments *in vitro* were conducted to verify ADAMTS9-AS1/ADAMTS9-AS2 restrains the proliferation, migration and invasion of LUAD cells.

## Material and Methods

### Microarray Data Analysis

Gene expression omnibus (GEO, http://www.ncbi.nlm.nih.gov/geo/), as an international public repository, is used for high-throughput microarray and next-generation sequence functional genomic data sets (24). In the present study, the gene expression profile, GSE130779, was downloaded from the GEO; GSE130779 contained eight LUAD tissues and corresponding paracancer tissues, in which lncRNAs and mRNAs were found expressed differentially by limma package analysis in R. All probes were converted into their corresponding official gene symbols according to the annotation information provided each platform.

### Differential Expression Analysis

The limma package in R was used to analyze the expression profile of the differentially expressed lncRNAs/mRNAs with the limited condition of │log_2_FC│ >1 and *P*-value  < 0.05. Then, R was employed to make a volcano plot to visualize the differentially expressed lncRNAs/mRNAs with the same condition of │log_2_FC│ >1 and *P*-value  < 0.05.

### Exploration of miRNAs Bound to ADAMTS9-AS1/ADAMTS9-AS2/ADAMTS9

miRNAs that bind to ADAMTS9-AS1 and ADAMTS9-AS2, respectively, were found through miRcode. The miRNAs were obtained by intersecting with Venn diagram; then TargetScan and miRDB were separately used to find targeted ADAMTS9 miRNAs, and then by intersecting with Venn diagram (http://bioinformatics.psb.ugent.be/webtools/Venn/), miRNAs were obtained. The intersection miRNAs obtained by the two Venn diagrams were intersected again to obtain the overlapping miRNAs, miR-144-5p and miR-150-3p. The network interaction diagram of ADAMTS9-AS1, ADAMTS9-AS2, miR-144-5p, miR-150-3p and ADAMTS9 were made by Cytoscape.

### Correlation Analysis Between ADAMTS9-AS1, ADAMTS9-AS2 and ADAMTS9

The correlation between ADAMTS9-AS1, ADAMTS9-AS2 and ADAMTS9 was analyzed by the Spearman algorithm. Subsequently, the association between the three genes was also verified in GEPIA. Through KMplotter, the guess that ADAMTS9-AS1/ADAMTS9-AS2 expression was related to the prognosis of LUAD patients was confirmed.

### Protein–Protein Interaction (PPI) Network

The first 50 genes positively or negatively related to ADAMTS9 were analyzed for Protein–Protein Interaction analysis. These 100 genes were introduced into the STRING (https://string-db.org/), among which the protein interactions network was observed through Cytoscape and then Cytoscape was used for mapping to obtain the protein interactions between 100 genes. With the hubba plug-in in Cytoscape, 10 hubgenes were obtained by degrees method. The Mcode plug-in in Cytoscape was used for module analysis with the condition of Degree Cutoff: 2; Node Score Cutoff: 0.2; K-core: 2; Max the Depth: 100; Cluster Finding: A Haircut.

### Functional Enrichment Analysis

FunRich is an open access, standalone functional enrichment and network analysis tool (25), which is used to analyze the functional enrichment of these 100 genes, including the cellular component, molecular function, biological process, and biological pathway with the restricted condition of (│log_2_FC│ >1, *P <*0.05).

### GSEA Enrichment Analysis

The gene enrichment analysis of GSEA version 4.1.0 was used to explore how the expression level of ADAMTS9 affect the gene set of biological pathway in patients with LUAD, so as to further probe the possible mechanism of ADAMTS9 and the development process of LUAD. According to the expression level of ADAMTS9, the tumor tissue samples in TCGA-LUAD were divided into low expression group (n = 257) and high expression group (n = 258). The hallmark gene sets obtained from the MsigDB database of GSEA was used as the reference gene sets, and each analysis was repeated 1000 times according to the default weighted enrichment statistics method (http://software.broadinstitute.org/gsea/index.jsp). In GSEA, gene sets with P <0.05 and false discovery rate (FDR) <0.25 were regarded the significantly enriched gene sets.

### Cell Culture and Transfection

The LUAD cell lines (PC9, NCI-H1975, NCI-H1299 and A549) and human normal cell lines (BEAS-2B) were obtained from American Type Culture Collection. Cells were prepared and maintained according to standard cell culture procedures. The LUAD cells were cultured in in Dulbecco’s modified Eagle’s medium (DMEM, Invitrogen) supplemented with 10% fetal bovine serum (FBS, Gibco) at 37°C in humidified atmosphere of 5% CO_2_. Prior to transfection, the cell culture medium was replaced with antibiotic-free medium. The pcDNA3.1, pcDNA3.1-ADAMTS9-AS1 (pcDNA3.1-AS1) and pcDNA3.1-ADAMTS9-AS2 (pcDNA3.1-AS2) vectors were transfected into A549 and NCI-1299 cell lines with lipo2000 (Invitrogen; Thermo Fisher Scientific, Inc.), according to the manufacturer’s protocol. Cells were transfected for 24–48 h.

### RNA Extraction and Quantitative Real-Time PCR (qRT-PCR) Assay

Total RNAs were extracted and purified with TRIzol reagent (Invitrogen) from cells according to the manufacturer’s instructions. qRT-PCR was applied to detect ADAMTS9-AS1/ADAMTS9-AS2 expression. RNA was reversely transcribed to a single-stranded cDNA with the help of a Reverse Transcription System Kit (Takara, Dalian, China). GAPDH was used as an internal control for ADAMTS9-AS1 and ADAMTS9-AS2 expression. Each experiment was performed in triplicate. All primers used are as follows: ADAMTS9: forward, 5’-ACTGTGTAGGACGTAGAATGA-3’; reverse, 5’-AAGCAGACCGTTGATGTT-3’; ADAMTS9-AS1: forward, 5’-CCATCACTAATCGCCAGGAT-3’; reverse, 5’-CTGTTGTGGAGTTGCCCTTC; ADAMTS9-AS2: forward, 5’-AAGAAACCCTGATGTCTGGCTGAA-3’; reverse, 5’-GTGTTACTTGAGGAGAAAGCGAAA; GAPDH: forward, 5’-GCTCTCTGCTCCTCCTGTTC-3’; and reverse, 5’-CGACCAAATCCGTTGACTCC-3’.

### CCK-8 Assay

The CCK-8 assay was performed to detect the proliferation of ADAMTS9-AS1 and ADAMTS9-AS2 using a CCK-8 kit according to the manufacturer’s instruction (Dojindo, Kumamoto, Japan). Cells (2 × 10^3^cells/well) were seeded into 96-well plates in 100 μl of culture medium and incubated for 24 h at 37°C. Then, 10 μl CCK8 reagent was added to each well at 24, 48 and 72 h. The optical density (OD) values with 450 nm were measured after 1 h (Thermo Fisher Scientific, Madrid, Spain). Each experiment was repeated three times.

### Scratch Wound Healing Assays

Scratch wound healing assays were performed to detect the horizontal migration of LUAD cells. Cells were seeded into 6-well plates until confluence. Then, the cells were washed with fresh medium without FBS and wounded by scratching a straight line with a sterile plastic tip. The migration images obtained with a microscope (40×) at 0, 24, 48 and 72 h after scratching. All experiments were repeated three times. The area in the blank was analyzed and quantified with the ImageJ software.

### Transwell Assays

Transwell assays were carried out to determine the migration and invasion ability of LUAD cells. Briefly, the cells (5 × 10^3^ cells/well) were seeded at the upper layer in basal medium without FBS, while lower chamber was filled with DMEM containing 10% FBS. After cells were incubated for 24 h, the cells remaining on the upper layer were removed gently using a cotton tipped swabs, meanwhile, those cells adherent to underside of the membrane were fixed with 4% paraformaldehyde for 30 min and stained with 0.1% crystal violet. The cells were counted and photographed by a microscope (40×) and quantified with the ImageJ software. Each assay was repeated three times.

### Western Blot Assays

The expression of vimentin, Snail1, Twist1, ZEB1 and Beta3 were detected by western blot assays. Cells were lysed with the protein extraction kit (Beyotime, Shanghai, China) and protein concentrations were measured by the BCA Protein Assay Kit (Sangon, Shanghai, China). Proteins (30 ug) were separated using 10% sodium dodecyl sulfate-polyacrylamide gel electrophoresis (SDS-PAGE) and transferred onto the polyvinylidene difluoride (PVDF) membrane (Abclonal, Wuhan, China). After blocked with 4% non-fat milk, the membranes were incubated with a 1:1,000 dilution of primary antibodies against vimentin, Snail1, Twist1, ZEB1 and Beta3 (Abclonal, Wuhan, China) at 4°C overnight. After 24 h, the membranes were washed thrice with TBST buffer and incubated with secondary antibodies at room temperature for 1 h. Protein expression levels were visualized using the chemiluminescence substrates (Millipore, Bedford, MA, USA). The integrated density of protein bands was quantified using Image Lab software (Bio-Rad, Hercules, CA, USA). GAPDH was used as an internal control. Each experiment was conducted in triplicate.

### Statistical Analysis

All data were presented as mean ± SD. Graph Pad Prism software (version 8; Graphpad Software, Inc.) was used to analyze all data by Student’s t-test or one-way ANOVA. The correlation coefficient of ADAMTS9-AS1, ADAMTS9-AS2 or ADAMTS9 was performed by Spearman or Pearson.

## Results

### Identified the Differentially Expressed lncRNAs and mRNAs

**Figure 1:**
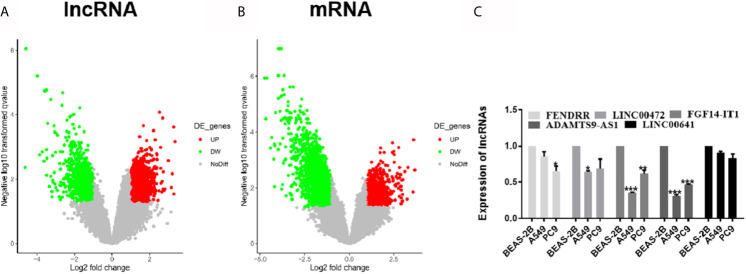
Differentially expressed of lncRNAs and mRNAs were identified. **(A)** Approximately 2,649 differentially expressed lncRNAs were screened, of which 1,660 ascended and 989 descended. **(B)** Approximately 4,303 differentially expressed mRNAs were screened, of which 1,128 ascended and 3,175 descended. **(C)** The five lncRNAs with the significant downregulation were selected for expression verification in LUAD cell lines and human normal lung epithelial cells. *p < 0.05; **p < 0.01; ***p < 0.001. LUAD, lung adenocarcinoma; lncRNA, Long non-coding RNA.

GSE130779 included eight patients with LUAD and corresponding paracancer tissues. From this, 2,649 differentially expressed lncRNAs were obtained in LUAD, among which 1,660 were up-regulated and 989 were down-regulated (**Figure 1A**). Likewise, there were 4,303 differentially expressed mRNAs, among which 1,128 were up-regulated and 3,175 were down-regulated (**Figure 1B**). The differentially expressed lncRNAs and mRNAs were visualized by volcano plot. The five lncRNAs (FENDRR, LINC00472, FGF14-IT1, ADAMTS9-AS1, and LINC00641) with the significant downregulation were selected for expression verification in LUAD cell lines and human normal lung epithelial cells. ADAMTS91-AS1 had the lowest expression in A549 and PC9 cell lines, and the down-regulated folds were 0.30 and 0.47, respectively (**Figure 1C**, *p < 0.05; **p < 0.01; ***p < 0.001). So ADAMTS9-AS1 was chosen as the object for subsequent analysis.

ADAMTS9-AS1 is the anti-sense of ADAMTS9. ADAMTS9 was found in the differentially expressed mRNAs of GSE130779, and was down-regulated in LUAD. The ADAMTS9 antisense lncRNA also includes ADAMTS9-AS2, which is also present in the GEO sequencing results and is low expressed in LUAD. To clarify whether there was a relationship between these three genes, their expression correlation was analyzed, and their positions obtained from the UCSC (https://genome.ucsc.edu/) were as follows: position of ADAMTS9 on chromosomes 3: 64,515,654–64,688,000 (Reverse strand −); position of ADAMTS9-AS1 on chromosomes 3: 64,561,322–64,592,757 (forward strand +); position of ADAMTS9-AS2 on chromosomes 3: 64,684,909-65,053,439 (forward strand +) (**Figure S1**).

### The Expression of ADAMTS9-AS1/ADAMTS9-AS2/ADAMTS9 Was Down-Regulated in LUAD

**Figure 2:**
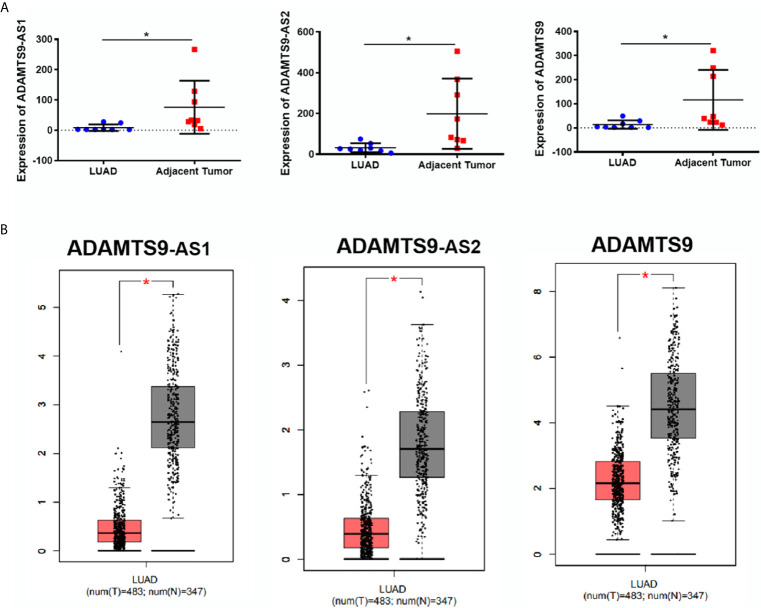
The expression of ADAMTS9-AS1/ADAMTS9-AS2/ADAMTS9 in LUAD tissues. **(A)** The expression of ADAMTS9-AS1/ADAMTS9-AS2/ADAMTS9 in LUAD tissues were analyzed by the GEO database. **(B)** The expression of ADAMTS9-AS1/ADAMTS9-AS2/ADAMTS9 in LUAD tissues were analyzed by the GEPIA database. *p < 0.05. LUAD, lung adenocarcinoma.

According to the expression profile data of ADAMTS9-AS1, ADAMTS9-AS2 and ADAMTS9 in the GEO database, the data showed that ADAMTS9-AS1, ADAMTS9-AS2 and ADAMTS9 are down-regulated in LUAD tissues (**Figure 2A**, *p < 0.05). At the same time, the down-regulated expression of ADAMTS9-AS1/ADAMTS9-AS2/ADAMTS9 in LUAD was confirmed by the GEPIA database analysis (**Figure 2B**). The results were consistent with the expression spectrum data in GEO.

### The Analysis of the Correlation Between ADAMTS9-AS1/ADAMTS9-AS2/ADAMTS9 Respectively

**Figure 3:**
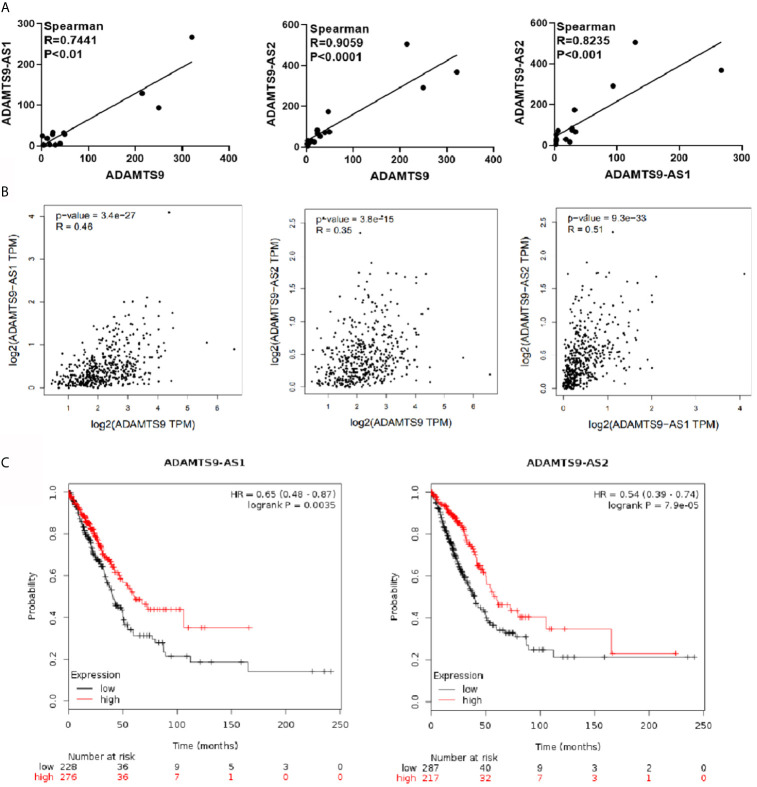
The analysis of the correlation between ADAMTS9-AS1/ADAMTS9-AS2/ADAMTS9 respectively. **(A)** The gene expression profile in the GEO database analyzes correlation analysis between ADAMTS9-AS1/ADAMTS9-AS2/ADAMTS9. **(B)** GEPIA verifies the correlation between ADAMTS9-AS1/ADAMTS9-AS2/ADAMTS9. **(C)** The prognosis of ADAMTS9-AS1 and ADAMTS9-AS2 was analyzed by a Kaplan–Meier Plotter (P < 0.01, P < 0.001).

Correlation between ADAMTS9-AS1, ADAMTS9-AS2 and ADAMTS9 were analyzed through gene expression profiles in GEO database. As shown in the **Figure 3A**, ADAMTS9-AS1 and ADAMTS9, ADAMTS9-AS2 and ADAMTS9, ADAMTS9-AS1 and ADAMTS9-AS2 are positively correlated, with Spearman correlation coefficients of 0.7441, 0.9059 and 0.8235, respectively, with p values less than 0.001. Then, we also verified it in the GEPIA database, in which the conclusion agreed with that in GEO database.(**Figure 3B**) Through Kaplan–Meier Plotter analysis, the result revealed that patients with high expression of ADAMTS9-AS1 and ADAMTS9-S2 had a good prognosis (**Figure 3C**, P < 0.01, P < 0.001). There is no statistical significance between ADAMTS9 expression and the survival rate.

### Identification of miRNAs Binging to ADAMTS9-AS1/ADAMTS9-AS2/ADAMTS9

**Figure 4:**
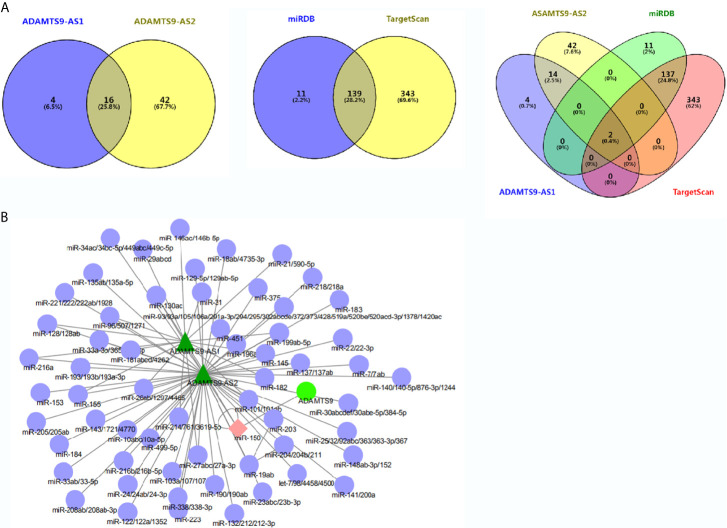
Overlapped differentially expressed genes from the intersection analysis. **(A)** Two miRNAs (miR-144-5p and miR-150-3p) combined with ADAMTS9-AS1/ADAMTS9-AS2/ADAMTS9 were analyzed. **(B)** The network interaction relationship of ADAMTS9-AS1/ADAMTS9-AS2/miR-150/ADAMTS9 is analyzed by Cytoscape.

MiRcode was used to analyze miRNAs which were bound to ADAMTS9-AS1 and ADAMTS9-AS2 respectively, among which 20 miRNAs were bound to ADAMTS9-AS1 and 58 to ADAMTS9-AS2. By Venn diagram, 16 overlapping miRNAs were obtained (**Figure 4A**). Subsequently, the miRNAs targeting ADAMTS9 were obtained by TargetScan/miRDB, with 150 and 482 miRNAs, respectively, and 139 intersected miRNAs were obtained by using Venn diagram (**Figure 4A**). By again intersecting the miRNAs obtained by the two Venn diagrams, miR-144-5p and miR-150-3p were obtained (**Figure 4A**). ADAMTS9-AS1/ADAMTS9-AS2/miR-150-3p/ADAMTS9 mutual effect net graph was made by Cytoscape (**Figure 4B**).

### The Correlation Analysis of Significant Genes Related to ADAMTS9

**Figure 5:**
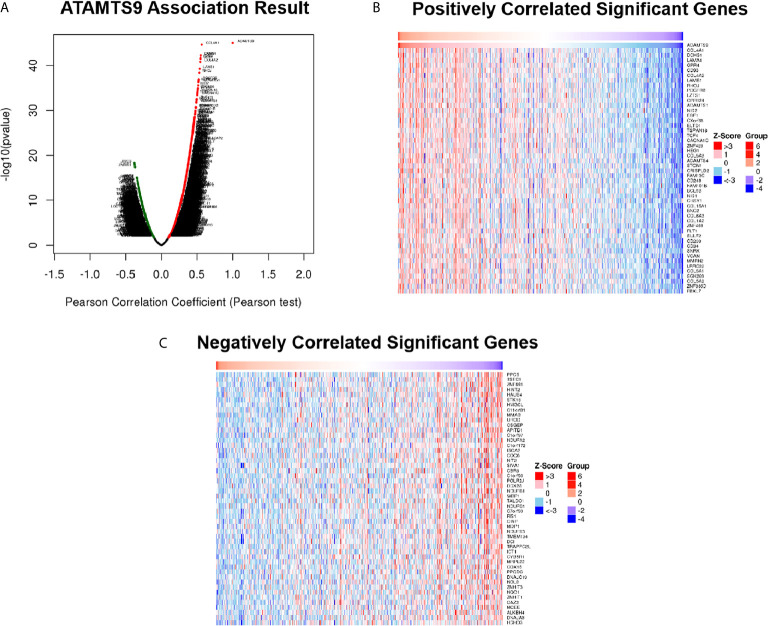
DEGs in association with ADAMTS9 in LUAD according to LinkedOmics. **(A)** The correlation between ADAMTS9 and genes differentially expressed in LUAD was evaluated by a Pearson test. **(B)** Heat maps showed the top-50 significant genes positively and **(C)** negatively correlated with ADAMTS9 in LUAD. The red stands for positively correlated genes and the blue stands for negatively correlated genes. LUAD, lung adenocarcinoma.

Differentially expressed genes associated with ADAMTS9 in LUAD were screened according to the LinkedOmics (**Figure 5A**). The correlation between ADAMTS9 and genes differentially expressed in LUAD was evaluated by Pearson test. Heat maps showed that the top-50 significant genes positively (**Figure 5B**) or negatively (**Figure 5C**) correlated with ADAMTS9 in LUAD (**Table 1**).

**Table 1:** Top 50 genes positively and negatively related to ADAMTS9.

	Positive correlation with ADAMTS9	Negative correlation with ADAMTS9
Gene Symbol	ADAMTS9, COL4A1, DCHS1, LAMA4, GPR4, CD93, COL4A2, LAMB1, RHOJ, PDGFRB, LZTS1, ADGRA2, ADAMTS1, NID2, EBF1, CXorf36, ELTD1, TSPAN18, TCF4, CACNA1C, ZNF423, HEG1, COL5A3, ADAMTS4, STON1, CRISPLD2, FAM13C, CD248, FAM101B, BCL6B, NID1, CHSY1, COL15A1, BNC2, COL6A3, COL1A2, ZNF469, FLT1, SULF2, CD200, CD34, SNRK, VCAN, MMRN2, LRRC32, COL5A1, PEAK1, COL5A2, ZNF385D, FBXL7	PPCS, TSTD1, ZNF691, HINT2, HAUS4, STK16, HMGCL, C11orf31, MMAB, UROD, OSGEP, APITD1, C1orf97, NDUFA2, C1orf172, ISCA2, COQ6, NIT2, SIVA1, CBR3, C1orf50, POLR2J, DDX28, NDUFB1, WBP1, TALDO1, NDUFC1, C7orf59, FIS1, CINP, MDP1, NDUFS3, TMEM134, DCI, TRAPPC2L, ICT1, CYB5R1, MRPL22, COX16, PPCDC, DNAJC19, NOL3, ZNHIT3, NQO1, ZNHIT1, OAZ3, MCEE, ALKBH4, DNAJA3, HDHD3

### Protein–Protein Interaction (PPI) Network Analyses

**Figure 6:**
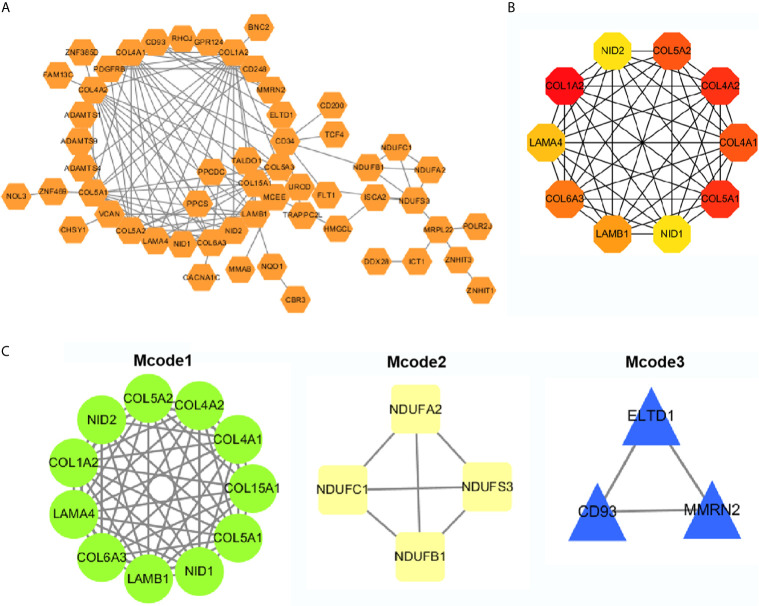
PPI network analysis. **(A)** PPI network analyses of the top 50 genes that positively and negatively correlated with ADAMTS9, and visualized with Cytoscape. **(B)** Ten Hub gene selection from the entire PPI network using the cytoHubba plug-in of Cytoscape software. **(C)** The Mcode plug-in in Cytoscape was used for moduled analysis, and finally three modules were obtained. PPI, Protein–protein interaction.

The top 50 genes related to ADAMTS9 plus and minus were analyzed for protein–protein interactions. These 100 genes were introduced into the STRING, and then Cytoscape was used for mapping to obtain the protein interactions between these 100 genes (**Figure 6A**). Using the hubba plug-in in Cytoscape, 10 hub genes were obtained (**Figure 6B** and **Table 2**). The Mcode plug-in in Cytoscape was used for module analysis, and finally three modules were obtained (**Figure 6C** and **Table 3**).

**Table 2:** The ten hub genes obtained.

Rank	Name	Score
1	COL1A2	16
2	COL4A2	15
3	COL5A1	15
3	COL4A1	14
5	COL5A2	14
6	COL6A3	13
7	LAMB1	12
8	LAMA4	10
9	NID2	9
10	NID1	9

**Table 3:** Module analysis.

Mcode	Gene
1	COL4A2, COL4A1, COL15A1, COL5A1, NID1, LAMB1, COL6A3, LAMA4, COL1A2, NID2, COL5A2
2	NDUFA2, NDUFS3, NDUFB1, NDUFC1
3	ELTD1, CD93, MMRN2

### Functional Enrichment Analysis

**Figure 7:**
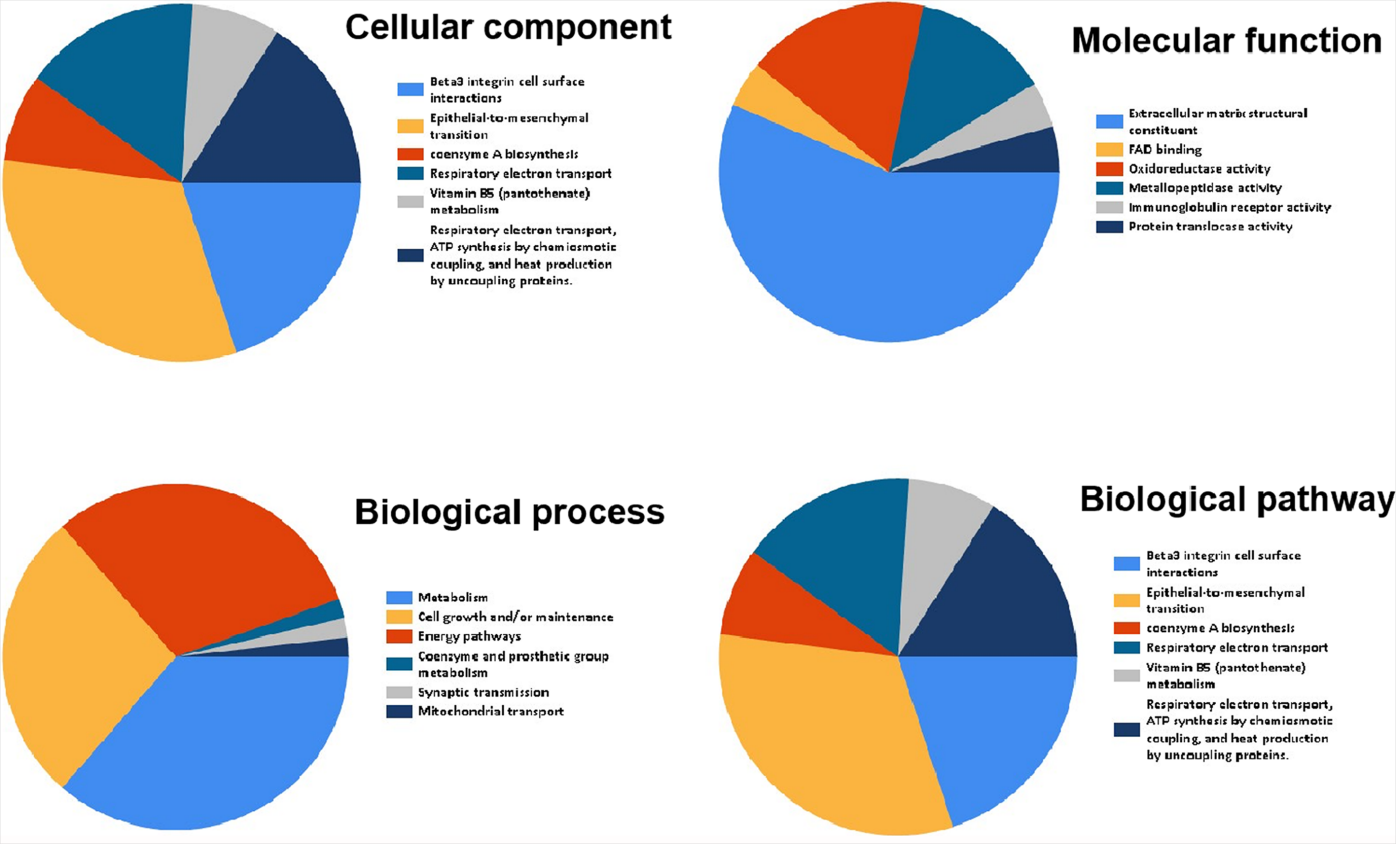
Go and KEGG pathway enrichment analysis of overlapped differentially genes.

Funrich was used for functional enrichment analysis. In terms of cellular component, these genes were mainly enriched in extracellular matrix collagen type V, mitochondrial respiratory chain complex I, basement membrane, extracellular space, extracellular (**Figure 7** and **Table 4**); With respect to molecular function, they were mainly enrich in extracellular matrix structural constituent, FAD binding, oxidoreductase activity, metallopeptidase activity, immunoglobulin receptor activity, transferase activity, transferring aldehyde or ketonic groups (**Figure 7** and **Table 4**); in terms of biological process, they were mainly enrich in metabolism, cell growth and/or maintenance, energy pathways, coenzyme and prosthetic group metabolism, synaptic transmission, mitochondrial transport (**Figure 7** and **Table 4**); in terms of biological pathway, they were mainly enriched in Beta3 integrin cell surface interactions, epithelial-to-mesenchymal transition, coenzyme a biosynthesis, respiratory electron transport, vitamin B5 (pantothenate) metabolism, respiratory electron transport, ATP synthesis by chemiosmotic coupling, and heat production by uncoupling proteins (**Figure 7** and **Table 4**).

**Table 4:** Functional enrichment analysis.

Gene enrichment	Terms	Percent of genes (100%)	P value
**Cellular component**	Extracellular matrix	13.33	<0.001
	Collagen type V	4.00	<0.001
	Mitochondrial respiratory chain complex I	5.33	<0.001
	Basement membrane	4.00	<0.001
	Extracellular space	12.00	<0.001
	Extracellular	28.00	<0.001
**Molecular function**	Extracellular matrix structural constituent	13.98	<0.001
	FAD binding	1.08	<0.01
	Oxidoreductase activity	4.30	<0.01
	Metallopeptidase activity	3.23	<0.05
	Immunoglobulin receptor activity	1.08	<0.05
	Transferase activity, transferring aldehyde or ketonic groups	1.08	<0.05
**Biological process**	Metabolism	21.51	<0.001
	Cell growth and/or maintenance	16.13	<0.001
	Energy pathways	18.28	<0.01
	Coenzyme and prosthetic group metabolism	1.08	<0.01
	Synaptic transmission	1.08	<0.05
	Mitochondrial transport	1.08	<0.05
**Biological pathway**	Beta3 integrin cell surface interactions	12.20	<0.001
	Epithelial-to-mesenchymal transition	19.51	<0.001
	coenzyme A biosynthesis	4.88	<0.001
	Respiratory electron transport	9.76	<0.01
	Vitamin B5 (pantothenate) metabolism	4.88	<0. 01
	Respiratory electron transport, ATP synthesis by chemiosmotic coupling, and heat production by uncoupling proteins.	9.76	<0. 01

### GSEA Enrichment Analysis

**Figure 8:**
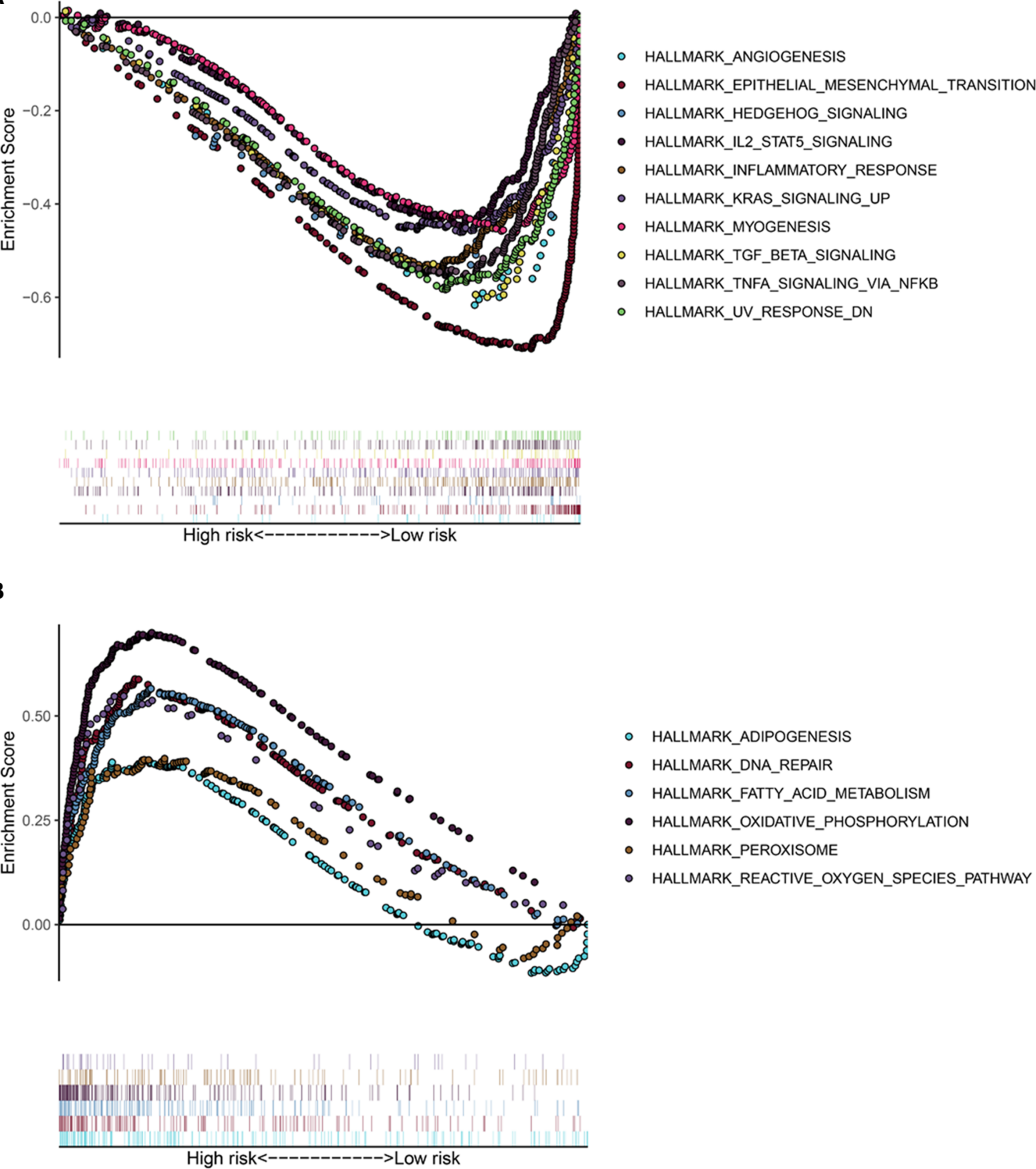
The gene set enrichment analysis (GSEA) of ADAMTS9. **(A)** GSEA showed that the samples with high ADAMTS9 expression. The gene sets with P < 0.05 and FDR < 0.25 were regarded as significantly enriched gene sets. **(B)** GSEA showed that the samples with low ADAMTS9 expression. The gene sets with P < 0.05 and FDR < 0.25 were regarded as significantly enriched gene sets. FDR, false discovery rate.

The TCGA-LUAD dataset was analyzed by GSEA method, and the influence of the expression level of ADAMTS9 on the gene set of various biological pathways was analyzed, and then the possible mechanism of ADAMTS9 promoting tumor occurrence and development was explored. Hallmark gene sets summarize and represent a specific clearly defined biological state or process. Using “Hallmark gene sets” as the reference gene set, it can be seen that the high expression samples of ADAMTS9 enrich the gene set (**Figure 8A**) related to angiogenesis, epithelial interstitial transformation, Hedgehog signal pathway, inflammatory response, KRAS signal pathway, IL-2, TGF- β and so on. ADAMTS9 low expression samples enriched adipogenesis, DNA repair, fatty acid metabolism, oxidative phosphorylation, peroxisome, reactive oxygen species pathway and other related gene sets (**Figure 8B**). These results suggest that ADAMTS9 may promote the occurrence and development of LUAD through angiogenesis, KRAS signal pathway, IL-2, TGF- β and other immune-related responses.

### Low Expression of ADAMTS9-AS1/ADAMTS9-AS2 in LUAD Cells

**Figure 9:**
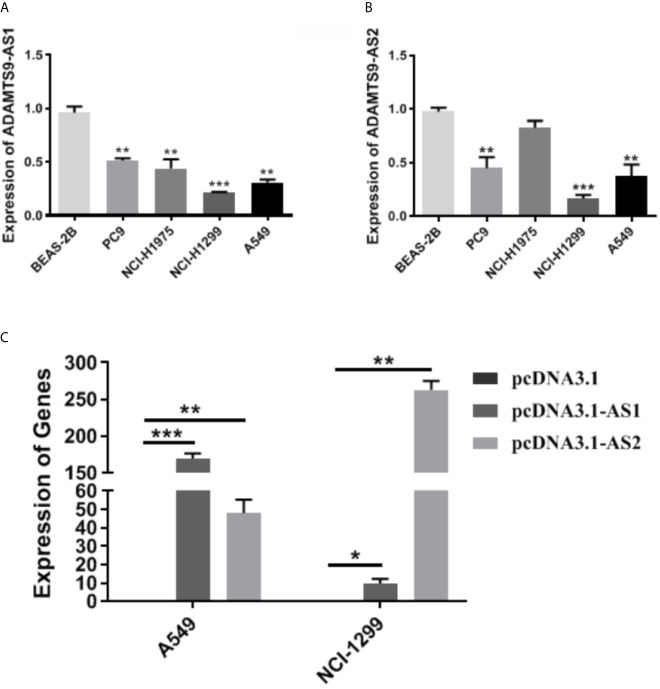
The expression of ADAMTS9-AS1 and ADAMTS-AS2 in LUAD cells. **(A)** The expression level of ASAMTS9-AS1 in LUAD cell lines. **(B)** The expression level of ASAMTS9-AS2 in LUAD cell lines. **(C)** The transfection efficiency of cells transfected with pcDNA3.1-AS1 or pcDNA3.1-AS2. *p < 0.05; **p < 0.01; ***p < 0.001. LUAD, lung adenocarcinoma.

The expression level of ADAMTS9-AS1/ADAMTS9-AS2 was detected by qRT-PCR. The results showed that the expression of ADAMTS9-AS1/ADAMTS9-AS2 is lower in A549 and NCI-H1299 cell lines than that in the other cell line (BEAS-2B, PC9 and NCI-H1975) (**Figures 9A, B**). NCI-H1299 and A549 were selected for subsequent cell function experiments. Moreover, the expression of lncRNAs (ADAMTS9-AS1 and ADAMTS9-AS2) increased in A549 and NCI-H1299 cell lines after the cells were transfected with pcDNA3.1-AS1 or pcDNA3.1-AS2 (**Figure 9C**, *p < 0.05; **p < 0.01; ***p < 0.001).

### ADAMTS9-AS1/ADAMTS9-AS2 Inhibits the LUAD Cells Proliferation, Migration and Invasion

**Figure 10:**
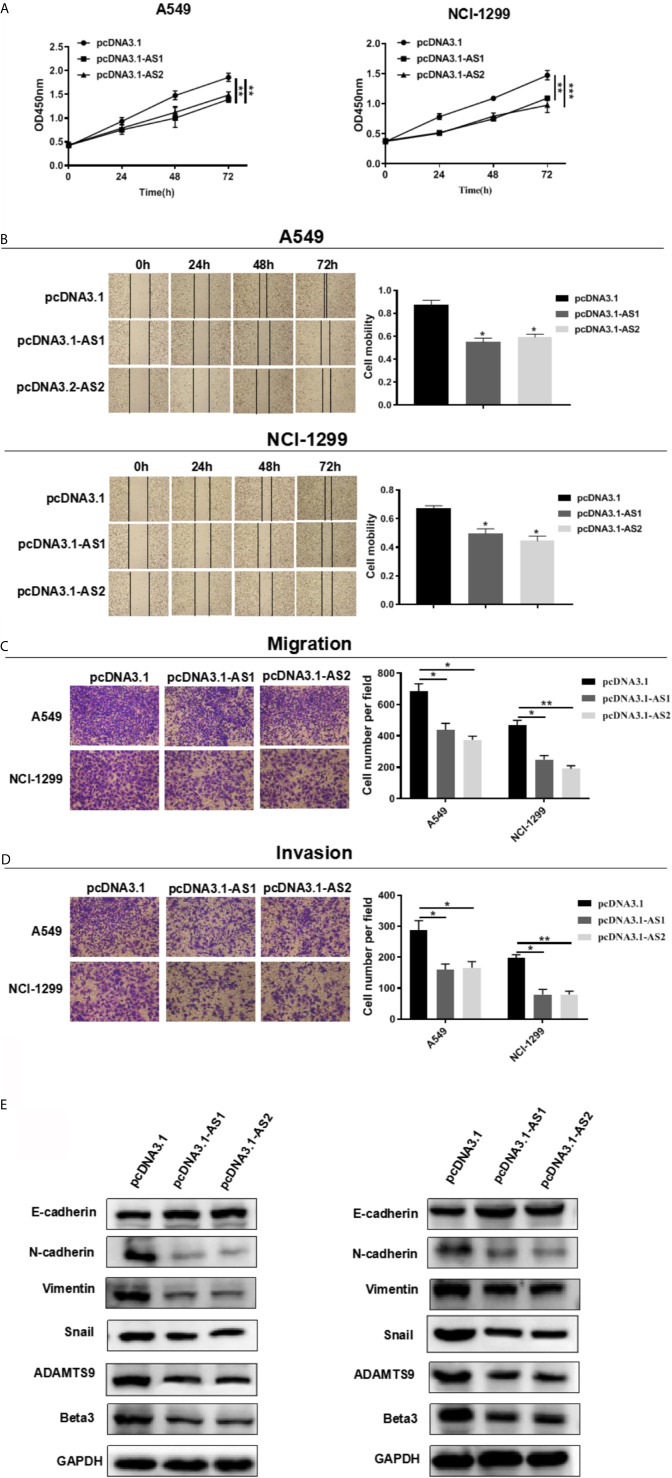
ADAMTS9-AS1/ADAMTS9-AS2 inhibits the LUAD cells proliferation, migration and invasion. **(A)** The proliferative ability of ADAMTS9-AS1/ADAMTS9-AS2 was detected by CCK-8. **(B)** The cell healing ability of ADAMTS9-AS1/ADAMTS9-AS2 was detected by a wound healing scratch assay. **(C)** The migratory ability of ADAMTS9-AS1/ADAMTS9-AS2 was detected by a transwell migration assay. **(D)** The invasion ability of ADAMTS9-AS1/ADAMTS9-AS2 was detected by a transwell invasion assay. **(E)** The influence of ADAMTS9-AS1/ADAMTS9-AS2 on Beta3 and EMT signal pathway detected by Western blot assays. *p < 0.05; **p < 0.01; ***p < 0.001. LUAD, lung adenocarcinoma.

We evaluated the role of ADAMTS9-AS1/ADAMTS9-AS2 in LUAD *in vitro*. Cell motility was determined using CCK-8, scratch wound healing and transwell assays. These results of cell proliferation experiment elucidated that the number of cells transfected with pcDNA3.1-AS1 or pcDNA3.1-AS2 were reduced (**Figure 10A**). In scratch wound heal assays, ADAMTS9-AS1 and ADAMTS9-AS2 over-expressed in A549 and NCI-H1299 cell lines had lower cell mobility than negative control group, which indicated ADAMTS9-AS1 and ADAMTS9-AS2 could inhibit LUAD cell horizontal migratory ability (**Figure 10B**). In addition, in cell transwell migration and invasion assays, ADAMTS9-AS1 and ADAMTS9-AS2 over-expressed in A549 and NCI-H1299 cell lines had lower ability of migration and invasion than negative control group, which indicated ADAMTS9-AS1 and ADAMTS9-AS2 could inhibit LUAD cell migration and invasion (**Figures 10C, D**). Functional enrichment analysis showed that enriched in Beta3 integrin cell surface interactions and EMT signaling pathways, ADAMTS9-AS1 and ADAMTS9-AS2 could regulate invasion and migration of LUAD cells via. Therefore, western blot assay was carried out to verify whether ADAMTS9-AS1 and ADAMTS9-AS2 affects LUAD cells invasion and migration *via* the Beta3 and EMT. The results indicated that overexpressed ADAMTS9-AS1 and ADAMTS9-AS2 inhibited the expression of vimentin, Snail1, Twist1, and ZEB1 mesenchymal markers while increased the expression of E-cadherin epithelial markers (**Figure 10E**), thus representing that ADAMTS9-AS1 and ADAMTS9-AS2 inhibited the Beta3 and EMT in LUAD cells.

## Discussion

LUAD is the most frequent histological NSCLC subtype. Currently, LUAD has become the most common lung cancer with increasing morbidity. Thus, it is urgently to identify suitable molecular markers for LUAD diagnosis and prognosis.

This study identified ADAMTS9-AS1, ADAMTS9 and ADAMTS9-AS2 by GSE130779 data set and GEO sequencing. Then, the low expression of ADAMTS9-AS1 and ADAMTS9-AS2 in LUAD tissues and cells was analyzed by the GEO database. Then ADAMTS9-AS1/ADAMTS9-AS2/ADAMTS9 were positively correlated with each other through the gene expression profile in GEO database. According to Kaplan–Meier plotter, the high expression of ADAMTS9-AS1 and ADAMTS9-AS2 had better prognosis. Next, through miRDB, miRcode and TargetScan, the miRNAs binding to ADAMTS9-AS1/ADAMTS9-AS2/ADAMTS9 were found, namely miR-150. The first 50 genes, positively or negatively correlated with ADAMTS9 were then found through LinkedOmics. Through functional enrichment analysis with Funrich, it was found that the genes were mainly enriched in Beta3 integrin cell surface interactions and EMT signaling pathways, which were associated with tumor invasion and migration. Studies had shown that MDSCs may promote the EMT and activate multiple signaling pathways in tumor cells, thus promoting tumor cell migration and distant metastasis (26–28). Finally, the results of cell experiments *in vitro* determined that ADAMTS9-AS1 and ADAMTS9-AS2 suppresses cells proliferation, migration and invasion in A549 and NCI-H1299 cell lines through the two signal pathways of Beta3 integrin cell surface interactions and epithelial-to-mesenchymal transition signaling pathways.

ADAMTS9-AS1 is an anti-sense transcript of the gene ADAMTS9. As previously reported, ADAMTS9-AS1 was closely associated with various cancers, including breast cancer (29), bladder cancer (30), colon adenocarcinoma (31). However, its role in LUAD remains unclear. In this study, patients with high expression of ADAMTS9-AS1 had better prognosis in survival analysis. Consistent with these results, ADAMTS9-AS1 was significantly down-regulated in LUAD tissues and cell lines. These results suggested that ADAMTS9-AS1 may function as tumor-suppressor in LUAD, and inspired us to speculate on the key role of ADAMTS9-AS2 in the progression of LUAD. In addition, ADAMTS9-AS1 was found to function as ceRNA, effectively sponging hsa-mir-96 and modulating the expression of PRDM16, thereby influencing tumor cell growth and proliferation in prostate cancer (32). In some extent, it supported our speculation that ADAMTS9-AS1 may play a ceRNA role in LUAD.

Likewise, as an anti-sense transcript of the gene ADAMTS9, ADAMTS9-AS2 was down-regulated in LUAD tissues by GEPIA analysis. Additionally, high expression of ADAMTS9-AS2 was associated with better prognosis. As reported in previous reports, ADAMTS9-AS2 could be regarded as potential predictive biomarkers of LUAD (33). Up-regulated ADAMTS9-AS2 suppressed progression of lung cancer (34). The above evidences indicated that ADAMTS9-AS2 played an important role in LUAD. Based on this result, we used functional assays to determine the roles of ADAMTS9-AS2 in LUAD, and the results implied that ADAMTS9-AS2 exerted as a tumor suppressor in LUAD.

Through bioinformatics analysis, we found miR-150-3p targeting ADAMTS9. In recent years, some researches have focused on the possibility of targeting miRNAs as part of cancer therapy (35, 36). miR-144-5p has been reported as a tumor suppressor in many cancers, including LUAD (37). A research revealed that high expression of miR-150-5p promoted the development of NSCLC by inhibiting LKB1 (38). Wang et al. validated that circLMTK2, as the sponge of miR-150-5p, promoted the proliferation and metastasis of gastric cancer. As for the validation of miR-150 targeting ADAMTS9, it remains to be performed (39).

## Data Availability Statement

Publicly available datasets were analyzed in this study. Thses data can be found here: [http://www.targetscan.org/], [http://mirdb.org/], [http://gepia.cancer-pku.cn/], [http://kmplot.com/analysis/], [http://funrich.org/index.html], [https://cytoscape.org/], and [http://www.gseamsigdb.org/gsea/index.jsp].

## Author Contributions

WL and WGL performed the experiments, analyzed the data, and wrote the manuscript. YC contributed to data acquisition and provided reagents. PZ contributed to the conception and design of the experiments, manuscript write up, and supervision of the study. LQ and WL contributed to the conception and design of the experiments, analysis and interpretation of data, manuscript write up, and supervision of the study. All authors contributed to the article and approved the submitted version.

## Funding

Anhui Provincal Natural Science Foundation [No.1808085MH266].

## Conflict of Interest

The authors declare that the research was conducted in the absence of any commercial or financial relationships that could be construed as a potential conflict of interest.

## Publisher’s Note

All claims expressed in this article are solely those of the authors and do not necessarily represent those of their affiliated organizations, or those of the publisher, the editors and the reviewers. Any product that may be evaluated in this article, or claim that may be made by its manufacturer, is not guaranteed or endorsed by the publisher.

## Supplementary Material

Supplementary Figure 1 The chromosome location of ADAMTS9, ADAMTS9-AS1 and ADAMTS9-AS2 in UCSC.
