# Supplementary figures and images for: Bioinformatics Analysis and Functional Verification of ADAMTS9-AS1/AS2 in Lung Adenocarcinoma
Source: Front Oncol. 2021 Jul 29;11:681777. doi: 10.3389/fonc.2021.681777 (PMC8358405; doi:10.3389/fonc.2021.681777)

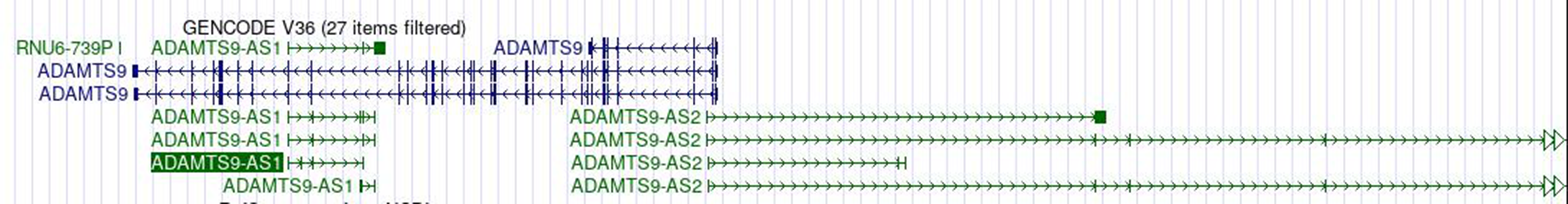

Supplement: Supplementary Figure 1 — The chromosome location of ADAMTS9, ADAMTS9-AS1 and ADAMTS9-AS2 in UCSC. [file Image_1.tif]
